# Calciphylaxis in POEMS syndrome: Case report

**DOI:** 10.1016/j.rare.2024.100019

**Published:** 2024-02-01

**Authors:** Danica Novacic, Thomas Uldrick, Alina Dulau-Florea, Colleen Evans Howe, Chyi-Chia R. Lee, Heidi H. Kong, William A. Gahl

**Affiliations:** a Undiagnosed Diseases Program, National Institutes of Health (NIH) Common Fund, Office of the Director, NIH, Bethesda, MD, USA; b Department of Laboratory Medicine, NIH Clinical Center, NIH, USA; c Medical Genetics Branch, National Human Genome Research Institute, NIH, USA; d Laboratory of Pathology, Center for Cancer Research, National Cancer Institute, NIH, USA; e Dermatology Branch, National Institute of Arthritis and Musculoskeletal and Skin Diseases, NIH, USA; f Vaccine and Infectious Disease Division, Fred Hutchinson Cancer Research Center, Seattle, WA, USA

**Keywords:** Calciphylaxis, POEMS, Myeloma, Case report

## Abstract

POEMS Syndrome is a constellation of findings including Polyneuropathy, Organomegaly, Endocrinopathy, Monoclonal plasma cell disorder, and Skin changes. Calciphylaxis, a microangiopathy involving vascular calcification and thrombotic occlusions, occurs rarely in POEMS. We present a case of prominent calciphylaxis that antedated the diagnosis of POEMS. The patient presented with extensive ecchymoses progressing to necrotic lesions in the setting of acute renal injury. Previously, she had chronic slowly progressive polyneuropathy, splenomegaly, hypothyroidism, amenorrhea, and ascites. Calciphylaxis was diagnosed on skin biopsy, and POEMS was diagnosed based upon clinical findings plus a bone marrow biopsy showing 15% lambda chain restricted plasma cells. Treatment for the calciphylaxis was supportive with fluids, tissue debridement, wound vacuum devices and antibiotics for secondary infection. Myeloma was treated with bortezomib and steroids. All aspects of the patient’s manifestations improved. We conclude that calciphylaxis can be a prominent feature of POEMS and can appear prior to recognition of the full-blown syndrome.

## Introduction

POEMS (Polyneuropathy, Organomegaly, Endocrinopathy, Monoclonal plasma cell disorder and Skin changes) is a rare paraneoplastic syndrome that occurs in association with lambda predominant monoclonal plasma cell proliferative disorder [1,2]. The clinical manifestations of POEMS can include polyneuropathy that is typically demyelinating and may be due to small nerve fiber edema, organomegaly that consists of hepatosplenomegaly and/or lymphadenopathy, and endocrinopathy that can involve the adrenal, thyroid, pituitary, gonadal, parathyroid, or pancreatic glands. The monoclonal plasma cell disorder consists of a monoclonal proliferation of plasmacytes that produces either non-functional fragments of immunoglobulin lambda light chains or light chains (almost always lambda type) bound to heavy chains with high usage of IgA heavy chain and, less commonly, IgG or IgM. Associated bone lesions are sclerotic in contrast to the classic lytic bone lesions associated with kappa light chain multiple myeloma. The skin changes reported in POEMS include hyperpigmentation, hypertrichosis, acrocyanosis, glomeruloid hemangioma, flushing and white nails.

Calciphylaxis is not widely recognized as part of POEMS. This severe, often catastrophic, microangiopathy causes endoluminal calcification that can be obliterative or superimposed with thrombotic occlusion of blood vessels [3]. It affects small arteries, arterioles, capillaries, and venules, usually in the subcutaneous fat but occasionally other organs. Calciphylaxis manifests clinically with the appearance of skin lesions that are painful, well-demarcated violaceous subcutaneous nodules or plaques that progress quickly into necrosis and ulcerations. Painful subcutaneous nodules without skin changes have also been described. This finding is classically described in patients with renal failure, secondary hyperparathyroidism and a high serum calcium-phosphate product.

We present a patient who developed calciphylaxis prior to her diagnosis of POEMS in the setting of only mild acute kidney injury, emphasizing the association between POEMS and calciphylaxis.

### Patient information

A 47 year-old Caucasian female was admitted to the NIH Undiagnosed Diseases Program (UDP) [4–6] and enrolled in a clinical protocol approved by the institutional review board of the National Human Genome Research Institute (https://clinicaltrials.gov/study/NCT00369421). The patient gave written, informed consent. Four years prior to our evaluation, she had presented with mild lower extremity edema and a persistent dry cough with clear rhinorrhea progressing over one year. She denied having asthma, allergies, cardiovascular or renal problems, fevers, sweats, purulent mucus, dyspnea, or malaise. She was not taking any medications, including nonsteroidal anti-inflammatory agents (NSAIDs) and was a lifelong non-smoker. She denied consuming excessive alcohol or illicit drugs. There was no family history of known genetic diseases.

### Clinical findings and timeline

At the time of her initial presentation, physical examination revealed a blood pressure of 155/100 with 1 + edema restricted to the lower extremities. Urinalysis showed mild proteinuria and hematuria. Serum creatinine was 1.2 mg/dL (normal, 0.5 – 1.0 mg/dL) and estimated glomerular filtration rate (eGFR) was 52 mL/min/1.73 m^2^ (normal >90 mL/min/1.73 m^2^). Serum albumin was normal. Anti-nuclear antigen (ANA), anti-neutrophil cytoplasmic antibody (ANCA), and anti-glomerular basement membrane antibody (anti-GBM) were negative, and serum complement (total, C3, and C4 components) was normal. Antihypertensive therapy was initiated.

Several months later, the edema progressed, and splenomegaly was noted. Chest x-ray showed mild congestion and pleural effusion. An echocardiogram was normal. Serum protein electrophoresis showed a monoclonal spike of 1.3 g/dL (normal, 0.7–1.2 g/dL) in the beta-1 region. Kappa and lambda light chains were both minimally elevated with a normal ratio. Quantitative immunoglobulins were normal. Beta-2-microglobulin was 4.3 mg/L (normal <1.7 mg/dL). A complete blood count was within normal limits. A bone marrow biopsy showed 8–10% plasma cells but no evidence of a plasma cell dyscrasia; monoclonal gammopathy of undetermined significance (MGUS) was diagnosed [7,8]. Cytogenetics and florescence in situ hybridization (FISH) were also negative for a myeloproliferative process. There was no hypercalcemia and there were no lytic bone lesions characteristic of multiple myeloma. A small sclerotic focus in the right femoral neck was considered an incidental enostosis.

During the ensuing 6 months, she slowly developed additional, seemingly unrelated symptoms. First, she noted amenorrhea; luteinizing hormone (LH) and follicular stimulating hormone (FSH) were mildly low, suggesting a diagnosis other than early menopause. The patient also noted a subtle generalized change in her skin tone. She appeared tan, but she had not had significant sun exposure. Adrenocorticotropic hormone (ACTH) was elevated at 150 pg/mL (normal, 0 – 46 pg/mL). The thyroid stimulating hormone (TSH) was 20 mIU/L (normal, 0.4 – 4.0 mIU/L) and L-thyroxine replacement was initiated. No other endocrinologic abnormality was found. The patient also began to develop mild paresthesia of the lower extremities; a neuropathy was confirmed by electromyography (EMG). A sural nerve biopsy showed mild-moderate loss of myelinated axons with a mild degree of ongoing axonal degeneration, confirming the peripheral neuropathy.

Over the subsequent 3 years, the edema slowly progressed to anasarca. A computed tomography (CT) of the abdomen and pelvis showed a large right pleural effusion, opacification of the right hemithorax, a small left pleural effusion, and massive ascites, which required paracentesis and thoracentesis twice per month. The fluid was transudative with negative cytology and cultures. Serum albumin was 1.5 g/dL (normal, 3.5 – 5.5 g/dL), but other liver function tests such as alanine aminotransferase (ALT) and aspartate aminotransferase (AST) were normal. Upper endoscopy and colonoscopy studies were normal. Liver imaging with CT was unremarkable, with only massive anasarca noted. A liver biopsy showed mild sinusoidal dilatation and mild portal and lobular chronic inflammation. The beta-2-microglobulin remained elevated and reached 7.2 mg/L; the highest creatinine was 1.5 mg/dL. A renal biopsy showed thrombotic microangiopathy, vascular injury with endothelial hypertrophy, mild intimal fibrosis, interstitial fibrosis, and tubular atrophy. Vascular endothelial growth factor (VEGF) was elevated at 693 pg/mL (normal, 31–86 pg/dL). Interleukin-6 (IL-6) was also elevated at 28 pg/mL (normal, 0.3–5 pg/mL).

**Fig. 1.**
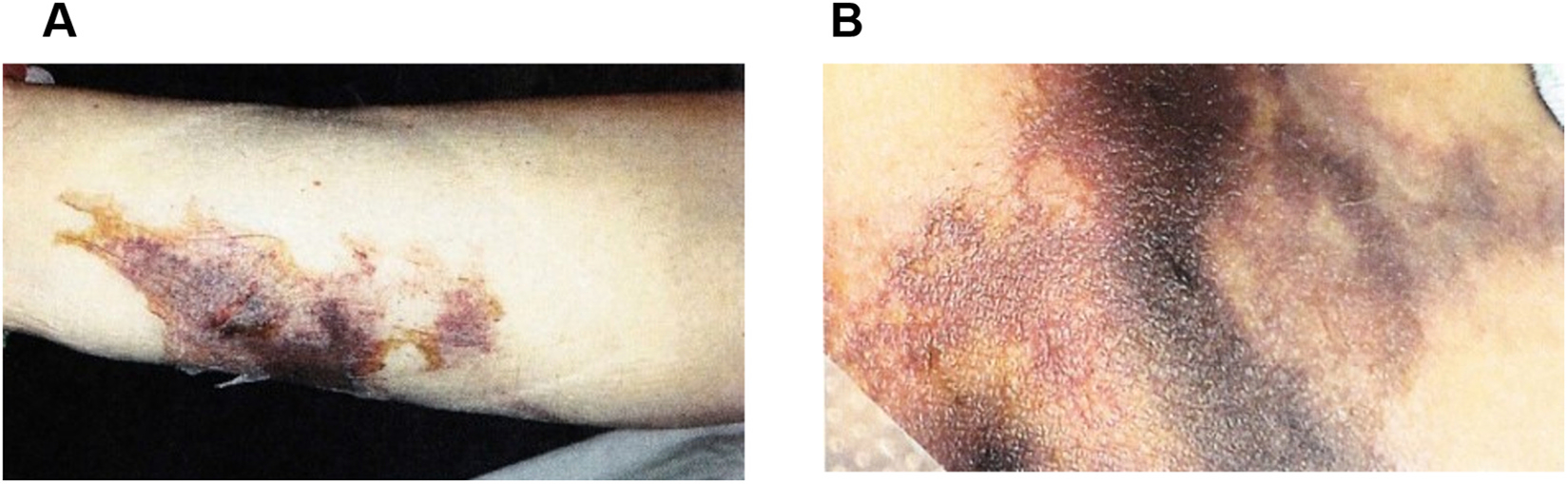
Skin findings. A. Distal lateral left lower extremity showing a necrotic skin lesion in a stellate pattern suggesting a vascular ischemic event. B. Left thigh calciphylaxis, with stellate necrotic skin lesion.

Independent of the subtle onset of mild hyperpigmentation of the skin, there was an acute onset of large areas of ecchymotic lesions (Fig. 1). She had multiple firm, deep, stellate necrotic plaques scattered on the trunk and extremities. These skin findings, along with acute renal injury and a serum creatinine of 3.3 mg/dL, prompted a 4-month hospitalization. Antibiotics were initiated, along with extensive deep tissue debridement; six separate vacuum-assisted closures of the wounds were performed. The renal injury responded well to intravenous fluids. The serum calcium concentration, corrected for hypoalbuminemia, was 10.4 mg/dL (upper limit of normal, 10.2). Hemoglobin was 9.2 g/dL (normal, 12 – 16 g/dL). The serum creatinine had peaked at 3.3 mg/dL but improved to normal. Phosphorous was not checked on admission labs but subsequently in the normal range and calcium-phosphate products never exceed 55.

### Diagnostic assessment

Considerations for a unifying diagnosis included a paraneoplastic syndrome with underlying malignancy, adaptive autoimmune dysregulation with formation of multiple autoantibodies, genetic somatic mutation of myeloid lineage cells causing innate auto-inflammatory dysregulation leading to increases in cytokines driving her symptoms, and other lymphoproliferative disorders such as multicentric Castleman disease (an angiofollicular lymph node hyperplasia with several histologic subclasses) or TAFRO syndrome (thrombocytopenia, ascites, bone marrow reticulin fibrosis, renal failure, and organomegaly). The normal gastrointestinal biopsies, including a Congo Red stain, ruled out amyloidosis.

**Fig. 2.**
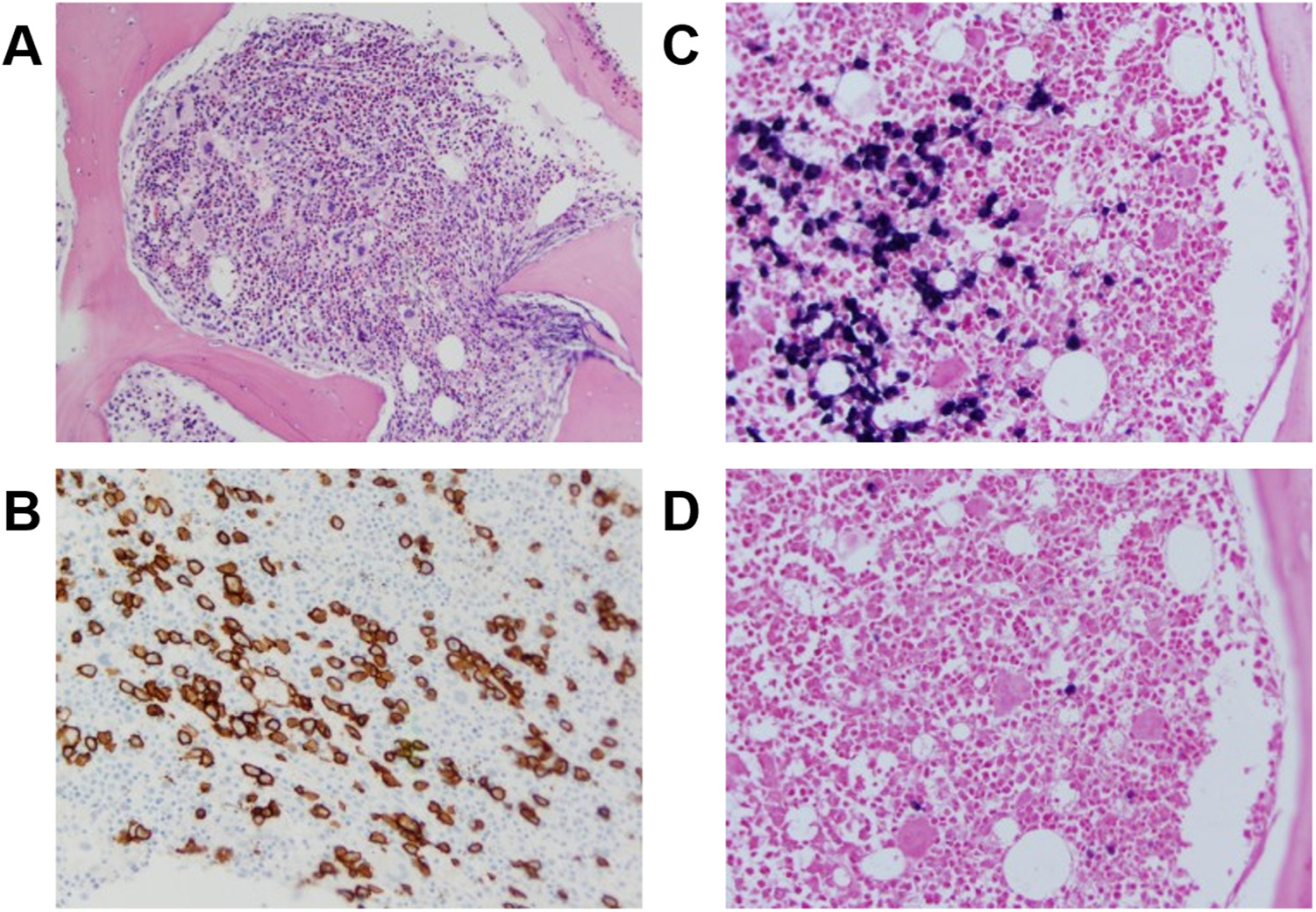
Bone marrow biopsy. A. Hematoxylin and eosin stain. B. CD138 + staining (brown) indicates 15% plasmacytes. C. Abundant lambda light chains stain dark purple. D. There is a paucity of dark purple staining for kappa light chains. (For interpretation of the references to color in this figure legend, the reader is referred to the web version of this article.)

A repeat bone marrow biopsy confirmed the diagnosis of multiple myeloma [9,10]; it showed reticulin fibrosis, atypical megakaryocytic hyperplasia, and hypercellularity (80%) with 15% plasma cells that were CD 138 + cells on immunohistochemical staining (Fig. 2 A,B). In situ hybridization (ISH) staining revealed lambda restriction, confirming the neoplastic nature of plasma cells and establishing a diagnosis of myeloma (Fig. 2 C,D) [4]. Flow cytometry showed a kappa:lambda ratio of 0.6%, with no monoclonal B lymphocytes or aberrant T-cell population.

The patient was referred to the NIH Undiagnosed Diseases Program [4–6], where a definitive diagnosis of POEMS was made based upon review of her various tissue biopsies as well her bone marrow findings. She was already significantly rehabilitated, with healed skin lesions, stable renal function, and no serositis.

**Fig. 3.**
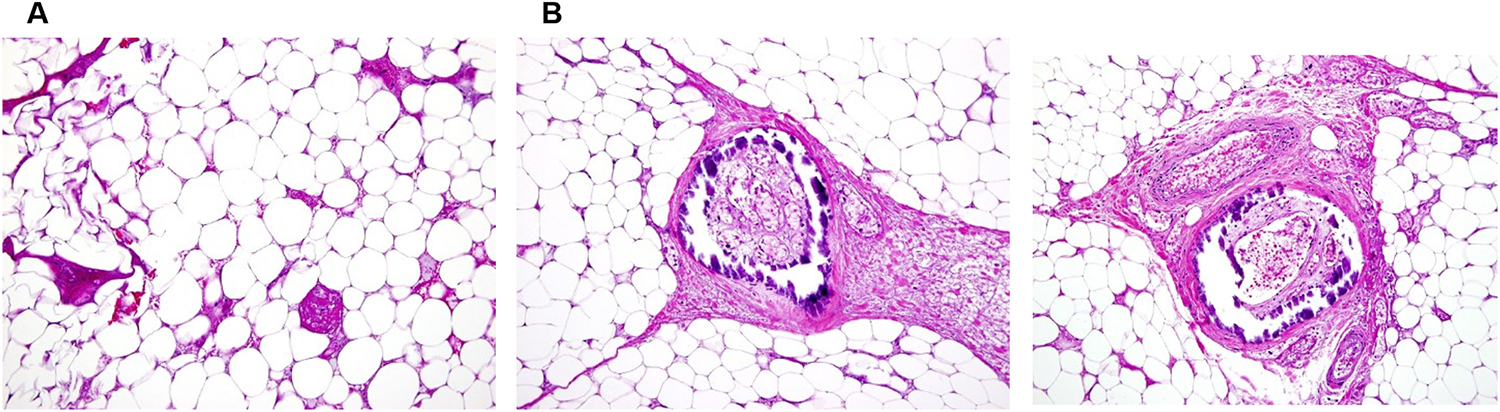
Thigh dermal biopsy. A. Subcutaneous adipose tissue with necrosis. B. Blood vessel with mural calcium deposition (dark purple) and thrombus in subcutaneous adipose tissue. The calcium deposits involve the intima and media of vessel walls of small and medium sized blood vessels.

Histologic confirmation of calciphylaxis was made based upon findings in the left thigh skin biopsy, which was described by the referring facility as showing intravascular fibrin thrombi in small subcutaneous vessels associated with prominent erythrocyte extravasation consistent with vascular occlusive disease. There was no inflammatory infiltrate or vasculitis. The adipose tissue contained fat necrosis with scarring (Fig. 3 A) and foci of dystrophic calcifications. Dermatopathology review at the NIH considered the necrotic skin lesions to represent calciphylaxis with adipose tissue containing occluded and calcified blood vessels in the subcutis. Several very small to medium-sized vessels had partially or completely occluded lumens with extensive calcification compatible with calciphylaxis (Fig. 3 B).

### Therapeutic intervention, follow-up, and outcomes

Chemotherapy was started with bortezomib [11] and corticosteroids. She was transferred to a rehabilitation facility and ultimately discharged to home. She completed the course of bortezomib. All of the patient’s symptoms rapidly improved. The serositis resolved, she did not require any additional paracenteses, her wounds healed completely, and her thyroid medication was reduced to a minimal dose. Renal function is stable. Serum VEGF continues to be in the low 200 range (normal, 31 – 86 pg/mL). Her laboratory results normalized (Table 1). A bone survey, performed after treatment and stabilization, showed no lytic lesions and only one sclerotic lesion at right femoral neck. The patient and has not relapsed in the past eight years. She continues to do well.

## Discussion

Beyond the classical features of POEMS – the polyneuropathy, organomegaly, endocrinopathy, monoclonal plasma cell disorder, and skin changes, additional features of POEMS have been reported [1,2]. Other described features of POEMS can include thrombocytosis, polycythemia, pulmonary hypertension, restrictive lung disease, diarrhea, papilledema, thrombotic diatheses, and low vitamin B12. Our patient with calciphylaxis underscores another less common manifestation of POEMS. POEMS can also be associated with Castleman’s disease, an angiofollicular lymph node hyperplasia with several histologic subclasses but generally causing cytokine dysregulation with high IL-6 levels, lymphadenopathy, and B symptoms such as fever, night sweats, and weight loss. Given the rarity of this disease and its less frequently occurring features, awareness of the potential manifestations of POEMS can facilitate more rapid diagnosis and treatment.

POEMS has been linked with a deregulation of cytokines, in particular a high serum or plasma VEGF state that is central to the symptomatology [12]. VEGF is secreted by many cells in response to anoxia and normally stimulates angiogenesis. Its effects include creating vascular fenestrations and thus increasing vascular leakage, migration and mitosis of endothelial cells, and chemotaxis for macrophages and granulocytes. Platelets and plasma cells are major sources of VEGF in POEMS syndrome. Excessive VEGF is also produced by malignant cells that are able to metastasize. The vascular leak mediated by VEGF is notable in POEMS since it can manifest as significant serositis, pleural effusions, edema, ascites and even axonal edema possibly contributing to the neuropathy.

Our patient exhibited a lambda-restricted predominant monoclonal gammopathy (MGUS) that later evolved to myeloma (Fig. 2), peripheral neuropathy, splenomegaly, high VEGF levels, recurrent pleural effusions, ascites with hypoalbuminemia and vascular leak, endocrine abnormalities with amenorrhea and hypothyroidism, and skin changes of mild hyperpigmentation but also multiple significant dermal ecchymotic lesions (Fig. 1). Aside from an incidental bony enostosis, our patient did not have sclerotic skeletal lesions often associated with lambda restricted myeloma.

Calciphylaxis is not noted in the diagnostic criteria for POEMS, although we identified several other case reports of calciphylaxis associated with POEMS [13–20]. Some have postulated that the high VEGF in POEMS promotes vascular injury and remodeling that leads to vascular calcifications [7]. Recently, high IL-6, lower serum albumin, more severe neuropathy and ascites were identified as factors associated with development of calciphylaxis in POEMS patients. In the same study, creatinine, calcium and phosphate levels were not associated with the development of calciphylaxis in POEMS patients, suggesting a different mechanism from that described classically in calciphylaxis and renal failure [18]. Our patient had evidence of cytokine dysregulation, including high VEGF and IL-6, neuropathy, low albumin, and severe ascites. These factors may have contributed to the development of calciphylaxis.

Calciphylaxis is a life-threatening disorder; mortality is 60–80%, with sepsis and internal organ failure being the leading cause of death. Prevention of calciphylaxis for end-stage renal patients on dialysis includes the use of a low phosphate diet, vitamin D analogs, and phosphate binders [16]. Treatment in the acute setting is supportive. The mainstay of therapy in survivors is aggressive wound care such as deep tissue debridement, hyperbaric oxygen treatments, and vacuum-assisted closure dressings [13]. In our patient, treatment of her underlying myeloma suppressed the toxic milieu and improved all of her symptoms, including those associated with calciphylaxis.

Several key points arise from this case. First, the findings of POEMS can be subtle, and the severity of POEMS symptoms does not correlate well with the myeloma burden. Recognition of the components of POEMS should alert the physician to the need for an in-depth search for underlying myeloma and vice versa; a patient with known myeloma should be followed for the development of POEMS features. Second, POEMS patients appear to have an increased risk of developing life-threatening calciphylaxis that may occur due to upregulation of inflammatory cytokines, ultimately leading to calcium deposition in vessels. Third, vascular changes from high VEGF may be a contributing factor in calciphylaxis in the absence of classically described lab evidence of high calcium-phosphate product. Finally, patients with POEMS and calciphylaxis may experience improvement in all of their symptoms after treatment for multiple myeloma, as in the case of our patient.

## Figures and Tables

**Table 1:** Laboratory results before and after treatment.

Lab	Pre-treatment	Post treatment	Normal Range

VEGF	693	199 (nadir)	31–86 pg/mL
IL-6	28	3.77	0.3–5 pg/mL
Beta-2-microglobulin	7.22	< 0.1	0.0–0.3 mg/L
Lambda light chains	5.01	1.73	0.57–2.63 mg/dL
Kappa light chains	2.8	1.42	0.33–1.94 mg/dL
Kappa:Lambda	0.55	0.82	1
Corrected calcium	8.68	9.6	8.9–10.1 mg/dL
Creatinine	3.3 (peak)	0.86	0.51–0.95 mg/dL
Anion gap metabolic acidosis	22	16	8–13
Bicarbonate	15	25	22–29 mmol/L
Albumin	1.5	4.3	3.5–5.2 g dL
Potassium	6.6	3.9	3.4–5.1 mmol/L
WBC	14.7 (left shift with 5%bands, 86% neutrophils)	7.73 (normal differential)	3.98–10.04 K/uL
Hemoglobin	13.2	16	11.2–15.7 g/dL
Platelets	160	237	173–369 K/uL
Parathyroid Hormone	Not checked	35	15–65 pg/mL
Adrenocorticotrophic Hormone	Not checked	150	0–46 pg/mL

Note: Phosphorous level was not checked with Calcium at time of calciphylaxis presentation. However, no subsequent Calcium × Phosphate product measurements exceed 55. VEGF, Vascular endothelial growth factor. IL-6, interleukin-6. WBC, whole blood count.

## Data Availability

No data was used for the research described in the article.
